# Impact of pH and temperature on the electrochemical and semiconducting properties of zinc in alkaline buffer media

**DOI:** 10.1039/c7ra12723e

**Published:** 2018-01-19

**Authors:** F. El-Taib Heakal, W. R. Abd-Ellatif, N. S. Tantawy, A. A. Taha

**Affiliations:** Chemistry Department, Faculty of Science, Cairo University Giza 12613 Egypt fakihaheakal@yahoo.com feltaibheakal@gmail.com +20 35727556 +20 1002449048; Faculty of Women for Arts, Science and Education, Ain Shams University Cairo 11566 Egypt

## Abstract

The electrochemical and semiconductive properties of spontaneously formed passive films on pure Zn were investigated in alkaline carbonate/bicarbonate buffer solutions as functions of pH and temperature. The study was performed in 0.1 M (CO_3_^2−^ + HCO_3_^−^) mixtures over the pH range 9.2 to 9.8 using open circuit potential, electrochemical impedance spectroscopy (EIS), potentiodynamic polarization and Mott–Schottky analysis techniques. Generally, zinc passivation is enhanced with either increasing pH or decreasing the ambient temperature. The steady state potential (*E*_ss_) value reveals that in pH 9.8 buffer the propensity of Zn for passivation is superior when compared with those in the other tested buffer solutions. The total surface film resistance (*R*_t_) derived from the impedance data proves this result, which is likely attributed to changes in composition and/or microstructure of the film. In pH 9.8 buffer solution the passivation tendency always decreases with temperature increase. However, in pH 9.2 the system behaves similarly up to 25 °C; afterwards zinc passivation trend was found to re-increase once more. The apparent activation energy for the corrosion process was evaluated and discussed. Analysis of Mott–Schottky plots was found to be suitable for characterizing the semiconductor properties of the naturally deposited barrier layers which are all consistent with the well-known n-type character of the oxide film on zinc. The absence of any evidences for the p-type semiconductive behavior indicates a preponderance of oxygen vacancies and zinc interstitials over metal vacancies. Moreover, Mott–Schottky results demonstrate that the donor concentration increases with either increasing pH or deceasing temperature commensurate with the increasing trends in the passive film thickness.

## Abstract 2

The electrochemical and semiconductive properties of spontaneously formed passive films on pure Zn in alkaline buffer solutions.

## Graphical abstract

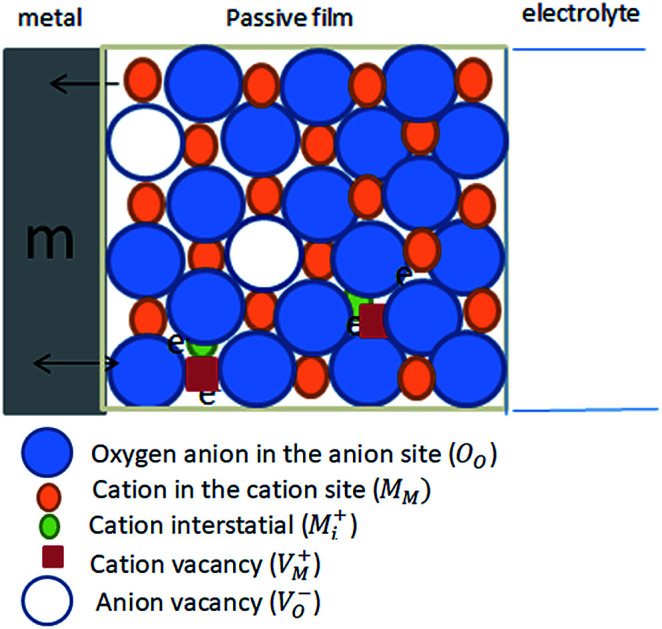

## 1. Introduction

Zinc (Zn) has been used for widespread applications in many industries such as electroplating and photo-catalytic processes to develop energy harvesting devices like solar cells.^1–3^ It is present in the earth crust as a mineral zincite (ZnO); however, most ZnO used commercially is formed synthetically. Zinc is commonly used as an anti-corrosion agent^4–6^ and in galvanization *e.g.* coating of iron or steel is the most familiar form. Zinc is used as the anode or fuel in the zinc–air battery fuel cell.^7^ The zinc–cerium redox flow battery also relies on a zinc-based negative half-cell.^8^ Zinc is divalent in all its compounds and compounds of Zn(i) do not naturally exist.^9^ It is one of the most commonly used battery electrode materials because of its low equilibrium potential, reversibility, compatibility with aqueous electrolytes, low equivalent weight, high specific energy, abundance, low cost, low toxicity and ease of handling.^10^ Moreover, zinc acts as a strong reducing agent and a moderately reactive metal. But in atmospheric air the surface of the pure metal can eventually forms a protective passive layer from the basic zinc carbonate, Zn_5_(OH)_6_(CO_3_)_2_,^11,12^ by reaction with carbon dioxide.

Zinc oxide (ZnO) is an inorganic compound usually appears as a white powder, nearly insoluble in water.^13^ It is widely used as an additive into numerous materials and products including; plastics, ceramics, glass, cement, lubricants, paints, adhesives, pigments, food (source of Zn nutrient), batteries, ferrites, fire retardants, *etc.*^14,15^ Zinc carbonate is of particular importance because it has been found to be responsible for the high corrosion resistance of zinc in atmospheric environments. The present study is focused on examining the electrochemical performance of zinc in alkaline carbonate/bicarbonate buffer solutions of pH 9.2 to 9.8. Electrochemical methods including electrochemical impedance spectroscopy (EIS) and potentiodynamic polarization measurements have been often used as useful tools to analyze the corrosion behavior of metallic materials.^16–18^ In alkaline solutions zinc corrosion occurs with oxide and/or hydroxide film formation. Thus, electronic properties and passive traits have to be also considered.^11,19,20^ For semiconductor materials, acquisition of Mott–Schottky analysis is a usual way for their electrochemical characterization. Mott–Schottky plot (inverse square of space charge layer capacitance (1/*C*_SC_^2^) *versus* semiconductor electrode potential (*E*)) gives doping density from the slope of the straight line and flatband potential from its intercept. The upper unfilled band in the electronic energy levels of a semiconductor is called the conduction band (*E*_c_) while the lower, almost filled band is called the valence band (*E*_v_). ZnO is an n-type semiconductor with a wide band gap energy (*E*_g_) of about 3.37 eV (368 nm) at room temperature^21^ and has high excitation binding energy of 60 meV and excellent chemical stability. Herein, the Mott–Schottky analysis for the passive film on pure zinc in alkaline carbonate/bicarbonate buffer electrolytes was also performed and the donor concentrations of its intrinsic defects were calculated as a function of both the forming solution pH and temperature.

## 2. Experimental

### 2.1 Materials and electrochemical methods

Zinc samples were cut from zinc rod of 99.99% purity as supplied from Johnson and Matthey (England). Each sample was welded to an electrical Cu wire and fixed with Araldite epoxy resin in a glass tube leaving a flat disc-shaped surface of 0.385 cm^2^ geometrical area for the zinc electrode. The electrodes were mechanically abraded successively using emery papers with finer grades 600, 800, 1200, 1500 grit to smoothing the electrode surface and obtain a mirror-like finish. The electrolytes used in the present investigation were naturally aerated carbonate/bicarbonate buffer mixtures (*x* ml 0.1 M Na_2_CO_3_ + *y* ml 0.1 M NaHCO_3_) as given in Table 1. Solutions with pH 9.2, 9.4, 9.5 and 9.8 were prepared using deionized water and analytical grade reagents, Applichem Panreac, Germany. A HANNA 213 pH meter with a combined glass electrode was used for the pH measurements. Electrochemical measurements were performed in the following sequence:

**Table 1:** pH values of (*x* ml 0.1 M Na_2_CO_3_ + *y* ml 0.1 M NaHCO_3_) buffer mixtures, with a pH range 9.2 to 9.8

pH	20 °C	9.2	9.4	9.5	9.8
	37 °C	8.8	9.1	9.4	9.5
*x* ml 0.1 M Na_2_CO_3_		10	20	30	40
*y* ml 0.1 M Na_2_HCO_3_		90	80	70	60

(i) Variation in open-circuit potential (OCP) of the working electrode under investigation was recorded relative to the silver–silver chloride (Ag/AgCl) reference electrode at different time intervals. Measurements were extended for 30 min until the electrode has achieved its steady state potential value (*E*_ss_).

(ii) Impedance measurements (EIS) were done at OCP using AC signal with amplitude of 10 mV over a frequency domain from 50 kHz down to 10 mHz.

(iii) After reaching the steady state potential, potentiodynamic polarization experiment was started using a scan rate of 1 mV s^−1^ over the potential range from −1.5 V to −0.6 V (*vs.* Ag/AgCl). Also, the corrosion current density (*i*_corr_) was calculated by Tafel extrapolation method from the linear parts of the cathodic and anodic branches.

(iv) Mott–Schottky measurements was carried out on the passive surface at a frequency of 3 kHz using a 10 mV AC signal and a step potential from −0.5 to 0.6 V, in the anodic direction. The flatband potential (*E*_FB_) of a semiconductor electrode can be experimentally determined by measuring the capacitance of the interface layer as a function of the applied potential. The measured capacitance (*C*) of the film/electrolyte interface can be described by the space charge capacitance (*C*_SC_) and the classical Helmholtz capacitance (*C*_H_) *via* the relation: 1/*C* = 1/*C*_SC_ + 1/*C*_H_. But, since *C*_SC_ ≪ *C*_H_, so the measured capacitance *C* value can be assumed to be that of the *C*_SC_ value when the potentials are applied at a sufficiently high frequency. Under this condition the interface surface film/electrolyte can be described by the Mott–Schottky equation:1
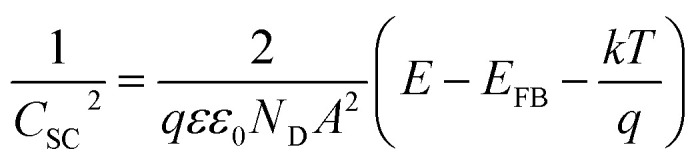
In this expression *C*_SC_ is the capacitance of the space charge region of the film at the applied potential *E*, *E*_FB_ is the flat-band potential, *q* is the charge of electron, *ε* is the dielectric constant of the semiconductor, *ε*_0_ is the permittivity of free space (8.854 × 10^−14^ F cm^−1^), *N*_D_ is the free donor concentration for the n-type semiconductor, *A* is the geometric surface area of the working electrode, *k* is Boltzmann constant and *T* is the absolute temperature. According to eqn (1) a plot of 1/*C*_SC_^2^*vs. E* is linear. The slope of this plot is commonly used to calculate the free carrier donors' concentration: 
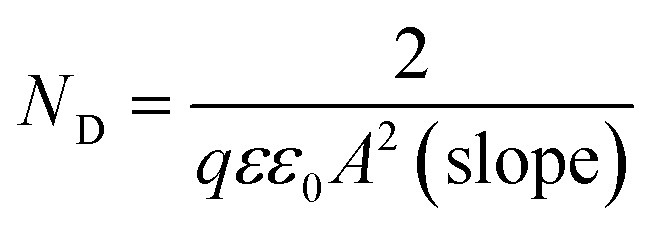
. The flatband potential can also be calculated from the intercept of the plot and its slope: 
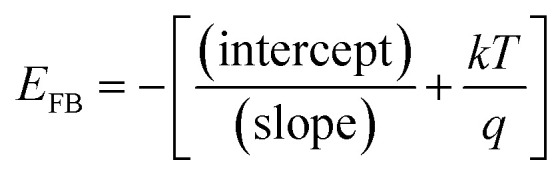
, where the factor (*kT*/*q*) can be neglected as it has a very small quantity of only 25 mV at room temperature (298 K).

All electrochemical measurements were carried out in a three-electrode cell array. The counter electrode was a large platinum sheet and all potentials were measured against a saturated Ag/AgCl reference electrode. Electrochemical measurements were performed using the electrochemical workstation IM6e Zahner-electrik, Meβtechnic (Kronach, Germany) which is controlled by a personal computer and Thales software.

### 2.2 Surface examination

The morphology of the surface films formed at different experimental conditions were observed using field emission scanning electron microscopy (FE-SEM), Quanta 250 FEG (Field Emission Gun). It is connected to EDX unit (Energy Dispersive X-ray Analyses), with an accelerating voltage 30 kV, magnification 14× up to 1 000 000, and resolution for gum 1 nm (FEI Company, Netherlands).

## 3. Result and discussion

### 3.1 Electrochemical properties

#### 3.1.1 Effect of pH

##### 3.1.1.1 Open circuit potential measurements

**Fig. 1:**
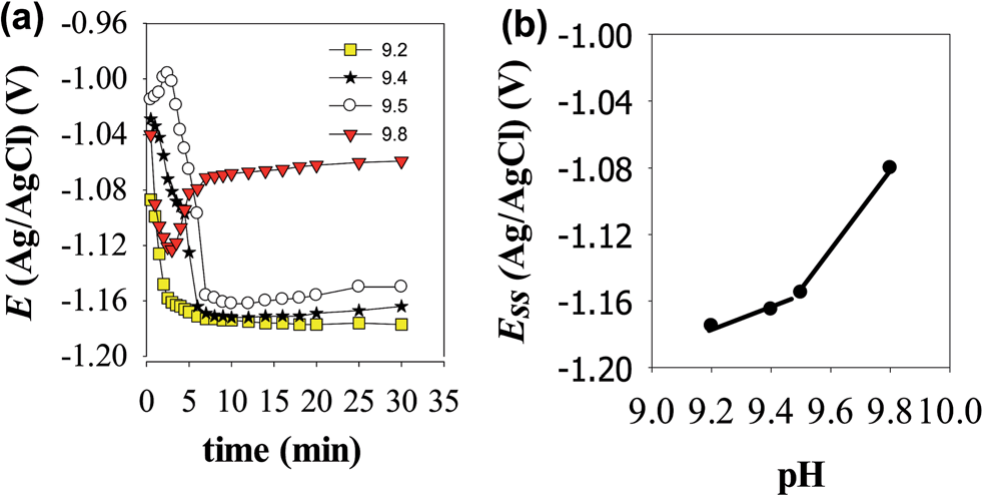
(a) Variation with time of the open circuit potential of Zn electrode in buffer solutions of different pH values at 25 °C. (b) Variation of the steady state potential (*E*_ss_) of Zn electrode with pH at 25 °C.

**Fig. 2:**
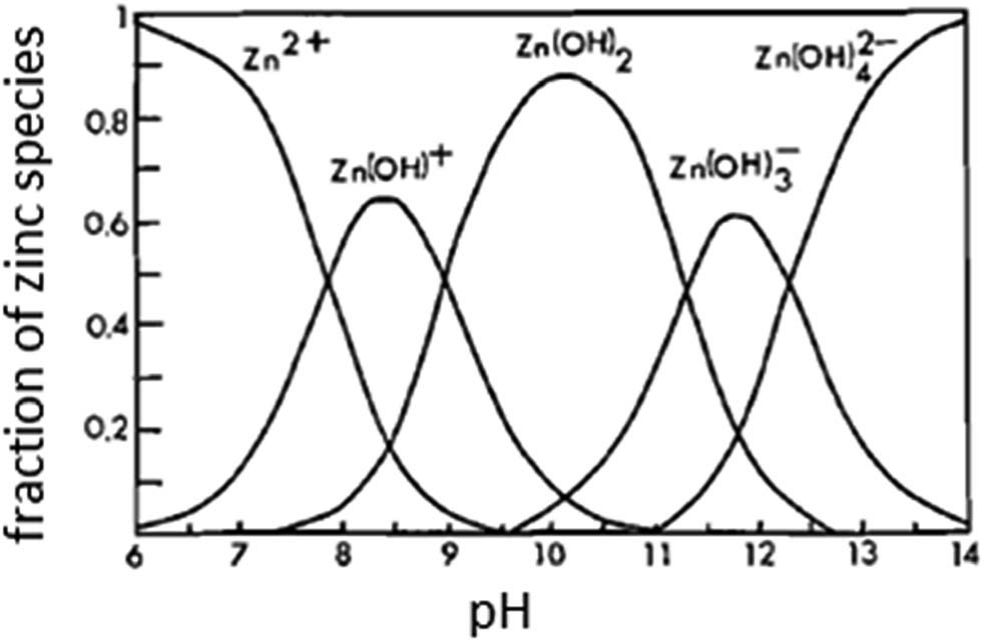
Distribution diagram for zinc species as a function of the solution pH at 25 °C.^16,17^

The open circuit potential (OCP) of Zn electrode was recorded over a period of 30 min in stagnant naturally aerated buffer solutions of pH 9.2, 9.4, 9.5 and 9.8. The potential of the working electrode under open-circuit conditions was measured separately against the reference Ag/AgCl electrode and the results of these measurements are presented in Fig. 1a. As it can be seen, in the two buffer solutions with lower pH values (9.2 and 9.4) the electrode potential drifts immediately with time in the active direction during the first 5 min then tends gradually to stabilize reaching its steady state value (*E*_ss_) after ∼10 min from the electrode immersion in solution. The negative shift in the potential indicates dissolution of the pre-immersed native film that formed naturally on the electrode surface. In pH 9.5 buffer solution Zn electrode experiences an incipient slight increase in its potential before the negative drift, indicating a limited growth in the native film that lasted only for 3 min. However, in the most basic buffer solution of pH 9.8 the electrode potential increases to more positive values after the initial fast decrease in its potential, implying that Zn surface tends to passivate with a higher rate at such higher pH value. Over the pH range 9.2 to 9.5, Fig. 1b shows that the steady state potential (*E*_ss_) increases to more noble values with increasing solution pH in accordance to the following linear relationship:

2 *E*_ss_ = *α* + *σ*pH

where *α* is the pH-independent steady state potential (*E*_ss_) which can be obtained by extrapolating the linear plot to pH = 0. The values of the slope (*σ*) for the linear relationship presented in Fig. 2b is very close to 0.064 and 0.250 V pH^−1^ for the first and second segments, respectively. Based on the Pourbaix diagram of Zn/H_2_O system,^22^ the slope of the first segment with its intercept (*α*) of −1.767 V can be taken as a qualitative criterion for a passivated zinc electrode in alkaline solutions. Nevertheless, the observed sharp increase in the slope at higher pH value (> 9.5) may be attributed to changes in the composition and nature of the formed film on the metal surface by changing the growth conditions at higher pH values.^23^ This will be further explored using the following AC and DC electrochemical results. Fig. 2 shows the species distribution diagram as calculated from the solubility data of Reichle *et al.*,^24^ and pertains to a saturated solution in equilibrium with the insoluble ZnO. It is observed that the predominant soluble zinc species in concentrated alkaline solutions is the tetrahedral ion (Zn(OH)_4_^2−^), but at pH 9.2 to 9.8, the predominant species at equilibrium is predicted to be Zn(OH)^+^ and/or Zn(OH)_2_, confirming that at OCP conditions passive zinc surface in this pH range is due to the presence of the equilibrium Zn(OH)^+^/ZnO and/or Zn(OH)_2_/ZnO by a dissolution–precipitation mechanism as will be indicated in the following section.

##### 3.1.1.2 EIS measurements

**Fig. 3:**
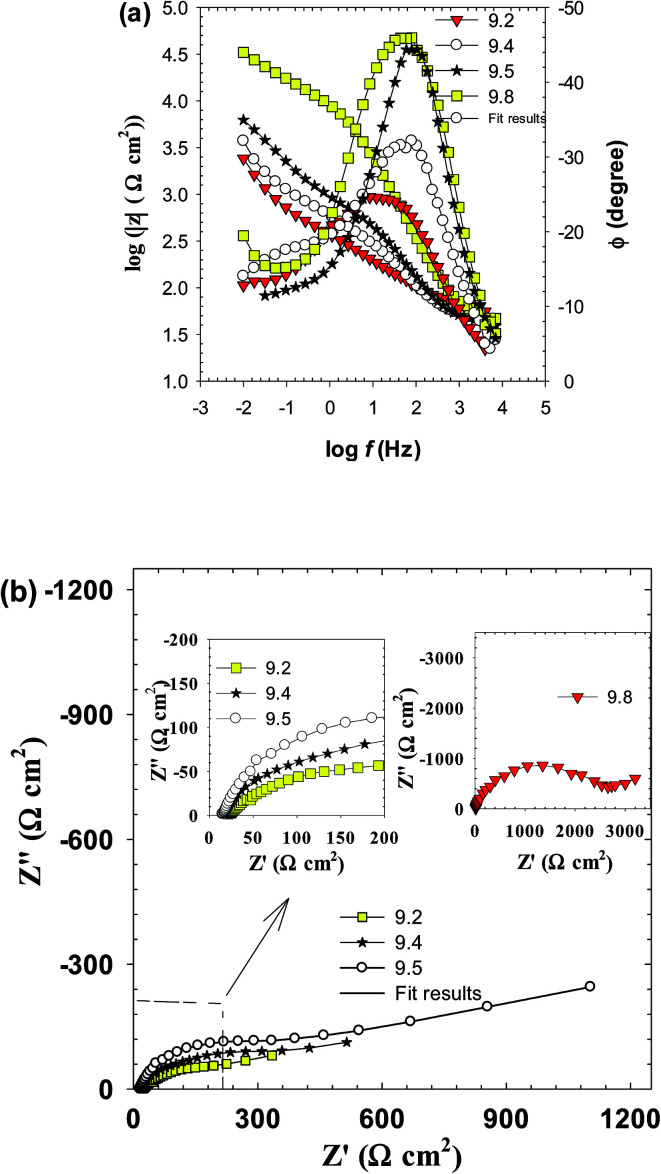
The impedance spectra of zinc electrode in carbonate/bicarbonate buffer solutions at 25 °C. (a) Bode plots at the pH range 9.2 to 9.8 and (b) Nyquist plots at pH 9.2, 9.4, 9.5 and the inset at pH 9.8.

Electrochemical impedance spectroscopy (EIS) is a non-destructive powerful technique for studying corrosion and passivation phenomena and can be used to probe in real time any differences in the electrode behavior due to surface modification.^25–28^Fig. 3a and b presents the impedance spectra of Zn electrode as Bode and Nyquist plots traced after 30 min from electrode immersion in the test buffer solutions of pH 9.2, 9.4, 9.5 and 9.8. Generally, as the pH increased, the Bode plots (Fig. 3a) display a continuous increase in the absolute impedance (|*Z*|) at the low to medium frequencies (10^−2^ to 10^4^ Hz). This is associated with a parallel significant increase in the phase angle maximum from −24.5° at pH 9.2 to −46.0° at pH 9.8, at the medium frequencies (10 to 10^2^ Hz) commensurate with a gradual shift in its value to lower frequencies. This behavior suggests a continuing enhancement in the corrosion resistance of the system. All these features demonstrate a gradual improvement in the protective nature and barrier property of the passive film formed spontaneously on the electrode surface as the solution pH is increased. In the meantime, each plot on the Nyquist format (Fig. 3b) exhibits linear region at low frequencies (LF) ended with a depressed semicircle capacitive loop at higher frequencies (HF). The observed time constants placed at high to medium frequencies are related to the capacitive response of the surface film, and the one appearing at low frequencies is due to the contribution of mass diffusion across this barrier film. All impedance spectra are similar except in the size of capacitive loops assigning the corrosion resistance of the barrier layer. This similarity implies that same mechanism occurring for the corrosion and passivation of zinc electrode at all pH values, albeit with different rates.

**Fig. 4:**
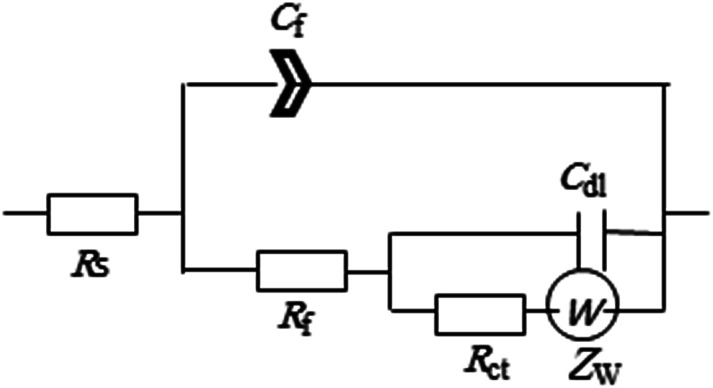
Two-time constant equivalent circuit used to fit the experimental EIS results.

To enable an accurate description for the electrochemical behavior of Zn in the tested alkaline buffer solutions from the obtained EIS data a special equivalent circuit (EC) model is needed. According to AC circuit theory, impedance spectra obtained for a given electrochemical system can be correlated to one or more equivalent circuit.^29^ Thus, different equivalent circuits were suggested to model the present data and the relevant model with the minimum number of electrical elements that gave the best fit with an average error of less than 0.8% to the experimental results is depicted in Fig. 4. This model consists from two time constants network comprising the solution resistance (*R*_s_) in serial connection with two parallel combinations representing the HF time constant due to the surface film capacitance (*Q*_f_) and its resistance (*R*_f_) and the LF time constant characterizing the double layer capacitance (*C*_dl_) and charge transfer resistance (*R*_ct_) together with the Warburg impedance (*Z*_w_). The existence of *Z*_w_ element suggests that the corrosion/passivation process for zinc in basic buffer solutions is controlled by diffusion phenomena.^30^ Due to surface heterogeneity of solid electrode resulting from surface roughness and formation of porous layers, a constant phase element (CPE) is commonly used to replace the capacitance (*C*) in real electrochemical process, which mainly depends on a non-ideal capacitance behavior.^27,30^ The impedance of the CPE is defined as:

3 *Z*_CPE_ = 1/*Q*(j*ω*^*n*^)

where *Q* in F cm^−2^ s^(*n*−1)^ is the general admittance function of CPE, which will be identical to the idealized capacitance of the passive film *C* in F cm^−2^ at angular frequency *ω* = 1 (*ω* = 2π*f* rad s^−1^),^25^*j* is the imaginary number 
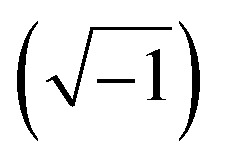
 and *f* in Hz or s^−1^ is the frequency of the applied AC signal during an impedance scan. The exponent *n* of the CPE is an empirical factor its deviation from unity is an indication of deviation of *Q* from ideal capacitance 0 ≤ *n* ≤ 1. Using Thales software provided with the workstation, fitting parameters of the equivalent circuit were estimated and listed in Table 2 as a function of pH of the tested buffer solutions.

**Table 2:** Equivalent circuit parameters for the impedance of zinc electrode as a function of the buffer solution pH at 25 °C

pH	*W* (kΩ s^0.5^ cm^2^)	*R*_ct_ (kΩ cm^2^)	*C*_dl_ (μF cm^−2^)	*R*_f_ (kΩ cm^2^)	*C*_f_ (μF cm^−2^)	*n*	*R*_S_ (Ω cm^2^)
9.2	0.09	0.012	5.35	0.156	8.91	0.762	8.7
9.4	2.43	0.033	4.64	0.419	8.38	0.886	10.7
9.5	3.09	0.101	3.40	0.581	8.0	0.875	13.4
9.8	5.07	0.580	0.21	7.510	7.84	0.883	19.0

As it can be clearly seen both film resistance (*R*_f_) and charge transfer resistance (*R*_ct_) increase with increasing solution pH, while their corresponding capacitance values *Q*_f_ and *C*_dl_ are decreased. Since the capacitance (*C*) is related to film thickness (*δ*) through the parallel plate expression:^31^

**Fig. 5:**
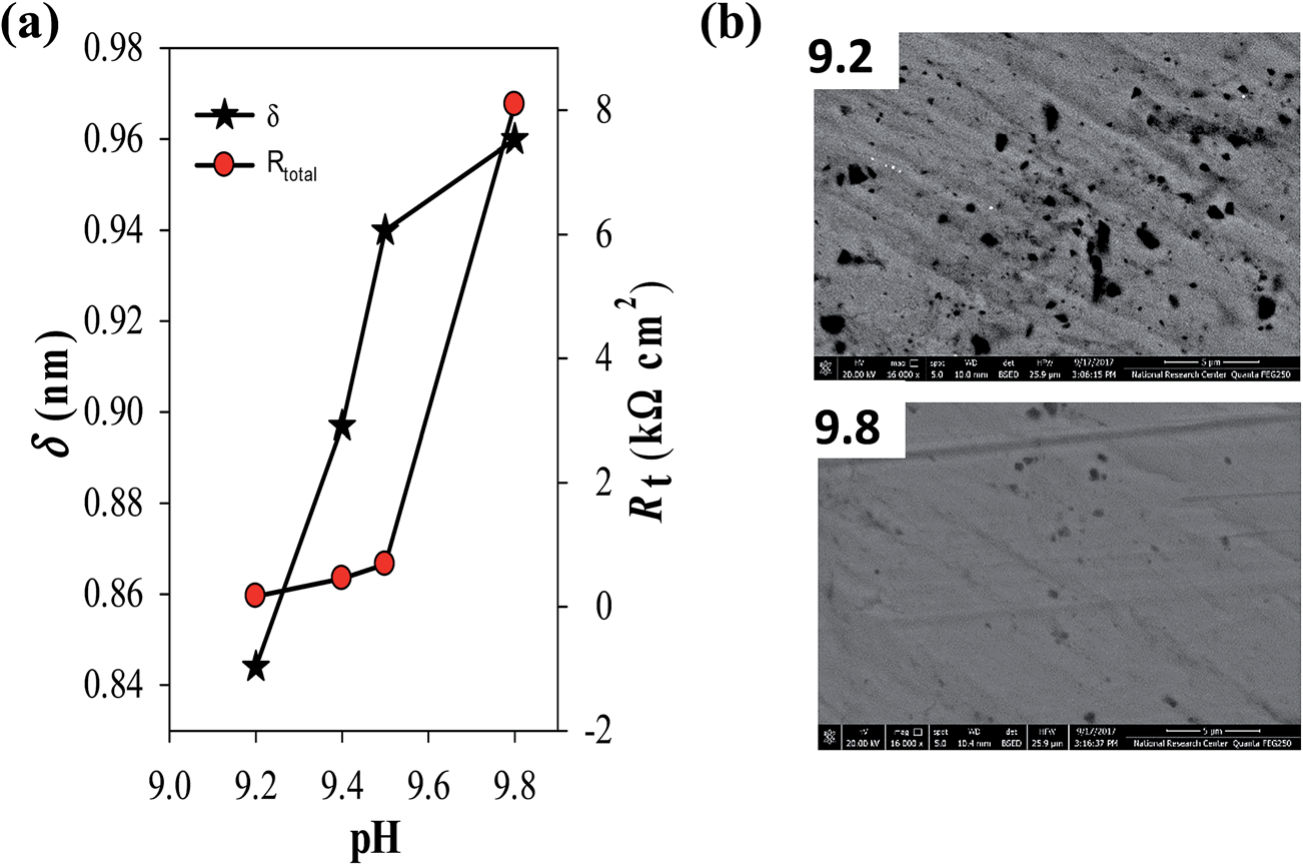
(a) Effect of solution pH on both the thickness (*δ*) and total resistance (*R*_t_) of the spontaneously formed passive film on pure zinc at 25 °C. (b) FE-SEM micrographs of zinc surface after 1 h immersion in carbonate/bicarbonate buffer solutions at 25 °C of pH (a) 9.2 and (b) 9.8.

4 *δ* = *ε*_r_*ε*_0_*A*/*C*

where *ε*_r_ is the relative dielectric constant of the passive film (*ε*_r_ = 8.5 for ZnO as in the literature),^32^*ε*_0_ is the permittivity of free space (8.854 × 10^−14^ F cm^−1^) and *A* is the geometric surface area (cm^2^) of zinc electrode. Therefore, passive film thickness can be easily calculated in nm to be correlated with the total resistance of the surface film (*R*_t_ = *R*_ct_ + *R*_f_). Fig. 5a demonstrates the development of these two parameters for the naturally grown passive film on zinc *vs.* the solution pH in the range 9.2 to 9.8. The plots reveal an increase in the film thickness (*δ* in nm) first with a higher rate until pH 9.5, then with a much sluggish rate after that. Meanwhile, the total film resistance value (*R*_t_ in Ω cm^2^) increases slowly at first until pH 9.5 and then rapidly afterwards. These results indicate that fast thickening rate of the surface film with increasing solution alkalinity leads to the formation of a passive layer with limited resistance, likely due to its defective porous nature. Whilst, the slow growth rate in the higher pH buffer solution sustain the formation of a film with compact structure having a significant higher resistance and better protective ability. In this context, the impedance results are in good agreement with the trend of the steady OCP values (*E*_ss_) depicted in Fig. 1b. SEM micrographs shown in Fig. 5b give further support to these results, where the passive film formed spontaneously on zinc surface in buffer solution of pH 9.2 is detected to have higher numbers of pores and voids in its microstructure when compared to the other one which was formed in pH 9.8 buffer solution, appearing to have a smoother and more perfect dense feature.

Concerning all these results, a simple plausible mechanism can be proposed based on the following steps. Thus, when zinc sample is immersed in naturally aerated carbonate/bicarbonate alkaline buffer solution two competitive processes can occur on its surface. The first is the partial dissolution of the native film, as well as the preferential dissolution of zinc atoms at the active anodic sites through the pores and defects in the surface,^33^

5 Zn → Zn^2+^ + 2e (anodic reaction)

6 2H_2_O + 1/2O_2_ + 2e → 2OH^−^ (cathodic reaction)

The second one is the growth of passive hydroxide/oxide film on the surface due to the following chemical reaction:

7 Zn^2+^ + OH^−^ → Zn(OH)^+^,

and/or

8 Zn^2+^ + 2OH^−^ → Zn(OH)_2_↓

where Zn(OH)_2_ is likely dehydrated to give ZnO as follows:^11^

9 Zn(OH)_2_ → ZnO↓ + H_2_O

Accordingly, zinc passivation in carbonate/bicarbonate alkaline buffer solutions operates through a dissolution–precipitation mechanism.^9^ This behavior indicates that buffer solution with higher pH value sustains the formation of a more resistive passive film on Zn surface with a better protective capability.

##### 3.1.1.3 Polarization measurements

**Fig. 6:**
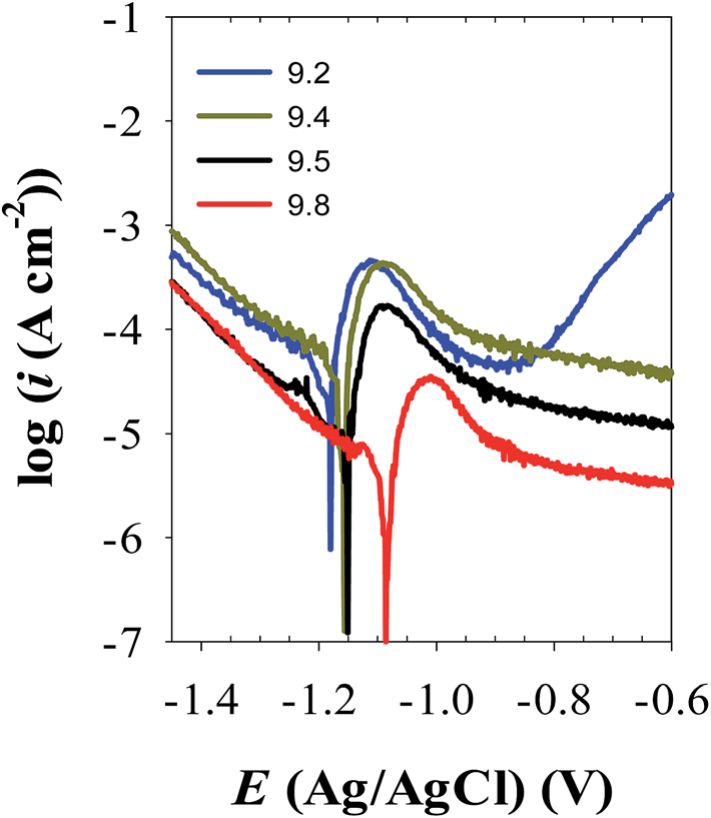
Potentiodynamic polarization curves of zinc electrode in buffer solutions with different pH values at 25 °C.

Fig. 6 presents the potentiodynamic polarization curves of zinc electrode in carbonate/bicarbonate buffer solutions of different pH values: 9.2, 9.4, 9.5 and 9.8 recorded at a scan rate of 1 mV s^−1^ over the potential range −1.6 to −0.6 V (*vs.* Ag/AgCl). As it can be seen, at all pH values an active–passive transition current peak is observed in the anodic trace which decreases and continuously shifts to more positive potential values with increasing solution pH from 9.2 to 9.8. This trend indicates that beyond the active range of potential, zinc exhibits self-passivation in all tested solutions which becomes more boosted at the higher pH.^4^ The transition from active to passive potential range is associated with low current density due to the formation of a protective ZnO film, which is likely considered to form directly from the metal rather than by precipitation from a super saturated layer of zincate near the surface in alkaline buffer media.^27^Table 3 shows the electrochemical corrosion parameters as estimated from the obtained polarization curves. The data correspond to the mean values of three different varieties tested at each condition. The values of the corrosion current density (*i*_corr_) were determined by Tafel extrapolation method.^34,35^ It can be noticed that the corrosion potential (*E*_corr_) shifts towards more positive values while the corrosion current density (*i*_corr_) decreases with increasing solution pH. This behavior points to an increase in the polarization resistance of the barrier passive layer on zinc surface as the pH of its contact electrolyte is increased.^20,27^ The results of polarization are in good consistency with the behavior of OCP and EIS measurements indicating that buffer carbonate/bicarbonate solution with higher pH value facilitates the formation of a more resistive passive film on Zn metal with better barrier protection.

**Table 3:** Polarization corrosion parameters of zinc electrode as function of the buffer solution pH at 25 °C

pH	*E*_corr_ (V_Ag/AgCl_)	*i*_corr_ (μA cm^−2^)	*β*_a_ (mV dec^−1^)	*−β*_c_ (mV dec^−1^)	*E*_peak_ (mV)	*i*_peak_ (A cm^−2^)
9.2	−1.179	45.6	44.4	311	−1.11	5.3 × 10^−4^
9.4	−1.157	42.2	52.4	238	−1.08	4.8 × 10^−4^
9.5	−1.152	30.6	37.8	262	−1.07	2.1 × 10^−4^
9.8	−0.996	29.4	33.6	126	−1.01	0.3 × 10^−4^

#### 3.1.2 Effect of temperature

It is of a prime importance to examine the influence of temperature on the electrochemical and semiconductor behavior of zinc metal in the tested buffer solutions. The aim is to explore any possible activation or passivation role on its traits. The two buffer solutions with higher and lower pH values, namely pH 9.8 and pH 9.2, were chosen as the test media to carry out the following series of experiments over the temperature range 5 to 45 °C.

##### 3.1.2.1 EIS behavior

**Fig. 7:**
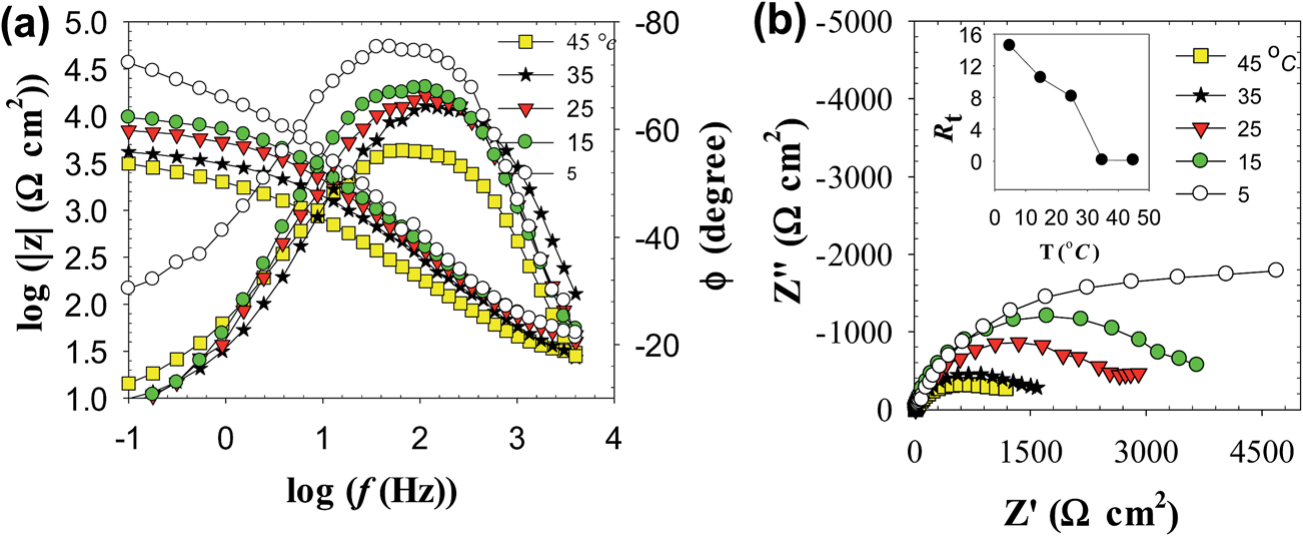
Impedance spectra of zinc electrode in pH 9.8 buffer solution at different temperatures (a) Bode plots and (b) Nyquist plots. Inset: variation of the total film resistance (*R*_t_) with *T*.

**Fig. 8:**
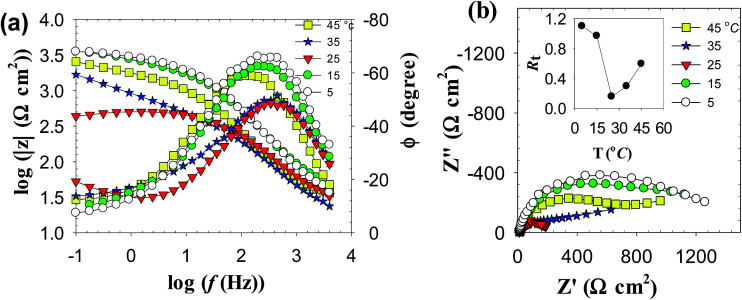
Impedance spectra of zinc electrode in pH 9.2 buffer solution at different temperatures (a) Bode plots and (b) Nyquist plots. Inset: variation of the total film resistance (*R*t) with *T*.

**Fig. 9:**
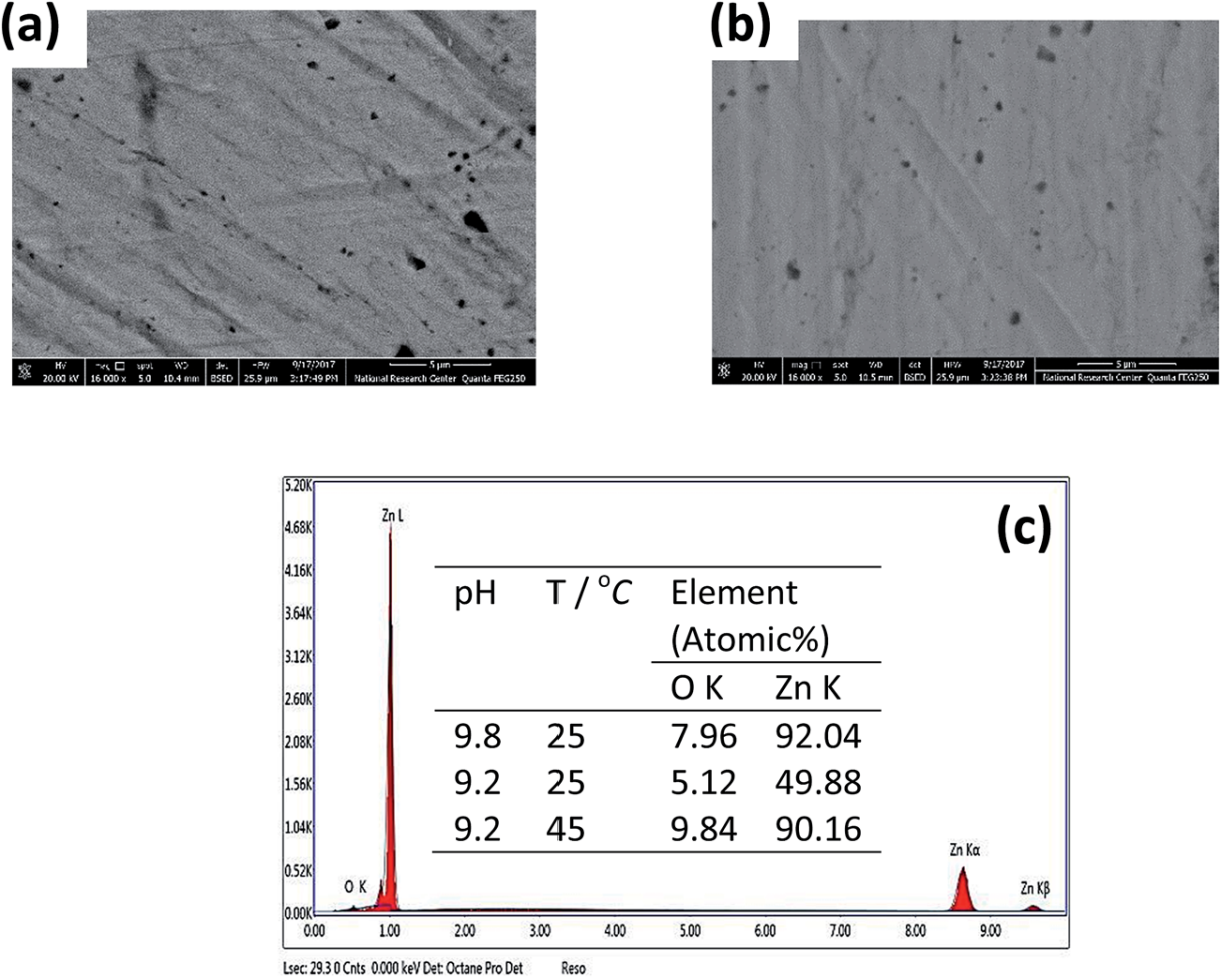
FE-SEM micrographs of zinc surface after 1 h immersion in carbonate/bicarbonate buffer solutions at pH (a) 9.2 at 45 °C and (b) 9.8 at 45 °C. (c) EDX spectrum of the surface passive film. Inset: elemental analysis of the passive film as a function of pH and temperature.

Fig. 7a shows the effect of temperature (*T*) on the Bode plots of zinc after 30 min immersion in carbonate/bicarbonate buffer solutions of pH 9.8 under the free corrosion conditions. It should be noted that all impedance responses have similar shapes characterized by a broad phase maximum observed in the *ϕ*–log *f* plot at the medium frequency (100 to 10 Hz). Furthermore, the spectra show a rather decreasing absolute impedance (|*Z*|) and phase maximum with raising temperature. The corresponding Nyquist plots of this data are presented in Fig. 7b, where a continuous reduction in the diameter of the depressed capacitive loop with temperature rise is observed. These results indicate that raising solution temperature has a negative effects on the passive film formed spontaneously on zinc surface immersed in pH 9.8 buffer solution. Fig. 8a and b reveals similar behavior for the tested zinc electrode in pH 9.2 buffer solution over the range 5 to 25 °C. However, at *T* > 25 °C a reverse trend is observed where the modulus |*Z*| and the phase maximum in the Bode plots (Fig. 8a), as well as the diameter of the capacitive loop in the Nyquist plots (Fig. 8b) are all increased once more with the temperature rise above 25 °C. This feature suggests that film formation–dissolution process for zinc in pH 9.2 buffer solution is likely shifted more towards formation at ambient *T* > 25 °C. SEM image shown in Fig. 9a provides a support for these traits where the surface of zinc sample is covered with intact and compact film after 1 h immersion in pH 9.2 buffer solution at 45 °C and becomes almost comparable to the featured surface film formed in pH 9.8 at 25 °C (Fig. 5b). Whilst for pH 9.8 film (Fig. 9b), raising the temperature to 45 °C produces adverse impact on its notable microstructure at 25 °C. In regard with the distribution diagram of zinc species at 25 °C (Fig. 2), it can be realized that at pH 9.2 the prevailing species is mainly Zn(OH)^+^. Raising the ambient solution temperature more than 25 °C can probably activate thermally the transformation of Zn(OH)^+^ to the stable species Zn(OH)_2_ in accordance with the following equation:

10 Zn(OH)^+^ + OH^−^ → Zn(OH)_2_↓

Eventually this would increase the passivation tendency of the surface film as found experimentally. In fact, our results do agree with the previously reported work by Dirkse and Timmer,^36^ who found that zinc corrosion rate decreased with increasing KOH concentration, and further the rate decreased in going from 25 °C to 44 °C. Analysis of the impedance spectra for zinc electrode in both solutions as a function of temperature were performed using the same EC model depicted in Fig. 4, and the obtained theoretical circuit parameters are listed in Table 4. Careful inspection of the data in this table reveals several interesting inferences:

**Table 4:** Equivalent circuit parameters for the impedance of zinc electrode immersed in buffers solutions of pH 9.8 and 9.2 at different temperatures

*T* (°C)	*W* (kΩ s^0.5^ cm^2^)	*R*_ct_ (kΩ cm^2^)	*C*_dl_ (μF cm^−2^)	*R*_f_ (kΩ cm^2^)	*C*_f_ (μF cm^−2^)	*n*	*R*_s_ (Ω cm^2^)
**pH 9.8**							
5	2.180	1.050	0.12	13.47	5.29	0.862	16.51
15	4.020	0.827	0.17	9.62	5.84	0.846	13.78
25	5.070	0.580	0.21	7.51	7.84	0.883	13.39
35	17.55	0.012	9.24	0.11	17.24	0.593	12.08
45	23.22	0.008	16.2	0.10	20.77	0.566	11.58

**pH 9.2**							
5	0.282	81.65	2.42	1.018	5.19	0.859	11.35
15	0.378	70.53	3.49	0.899	5.78	0.824	13.82
25	0.894	0.012	5.35	0.156	8.91	0.762	8.73
35	0.182	0.024	2.7	0.296	9.74	0.683	6.09
45	0.271	0.048	2.42	0.595	10.57	0.816	12.1

(a) In pH 9.8 buffer solution, raising the ambient temperature decreases gradually the values of both charge transfer resistance (*R*_ct_) and film resistance (*R*_f_). This implies a decrease in the total surface resistance (*R*_t_ = *R*_ct_ + *R*_f_) with temperature as shown in Fig. 7b inset.

(b) A reverse trend is observed for the two corresponding capacitance values double layer capacitance (*C*_dl_) and film capacitance (*C*_f_). Where *C*_dl_ increases from 0.12 μF cm^−2^ at 5 °C and achieving a value of 20.77 μF cm^−2^ at 45 °C, which means a substantial thinning in the passive layer at elevated temperatures. The nearly more than 170 times decrease in the passive layer thickness leads to significant reduction in its protective ability at higher temperature.

(c) The impedance data for zinc in pH 9.2 buffer solution exhibit also similar trend over the temperature range 5 to 25 °C. But, at *T* > 25 °C the charge transfer resistance (*R*_ct_) remains almost unchanged while the *R*_f_ value increases from 156 Ω cm^2^ at 25 °C to 595 Ω cm^2^ at 45 °C as proven from Fig. 8b inset.

(d) In the meantime, the film capacitance value (*C*_f_) continues to increase over the whole tested temperature range 5 to 45 °C. These results point out to a possible change in the film microstructure which boosts its resistance at higher temperatures (> 25 °C). The SEM image shown in Fig. 9a and b manifests that the passive film formed on zinc sample after one hour immersion in pH 9.2 buffer solution at 45 °C becomes smoother and less defective when compared with the image of the film formed at 25 °C (Fig. 5b). This proves that raising the temperature of this buffer solution above 25 °C aids for forming a thin passive film with a coherent microstructure and better protective effect. For example, the resistance (*R*_f_) of this film at 45 °C is enhanced by more than three times relevant to its value at 25 °C.

##### 3.1.2.2 Activation energy

**Fig. 10:**
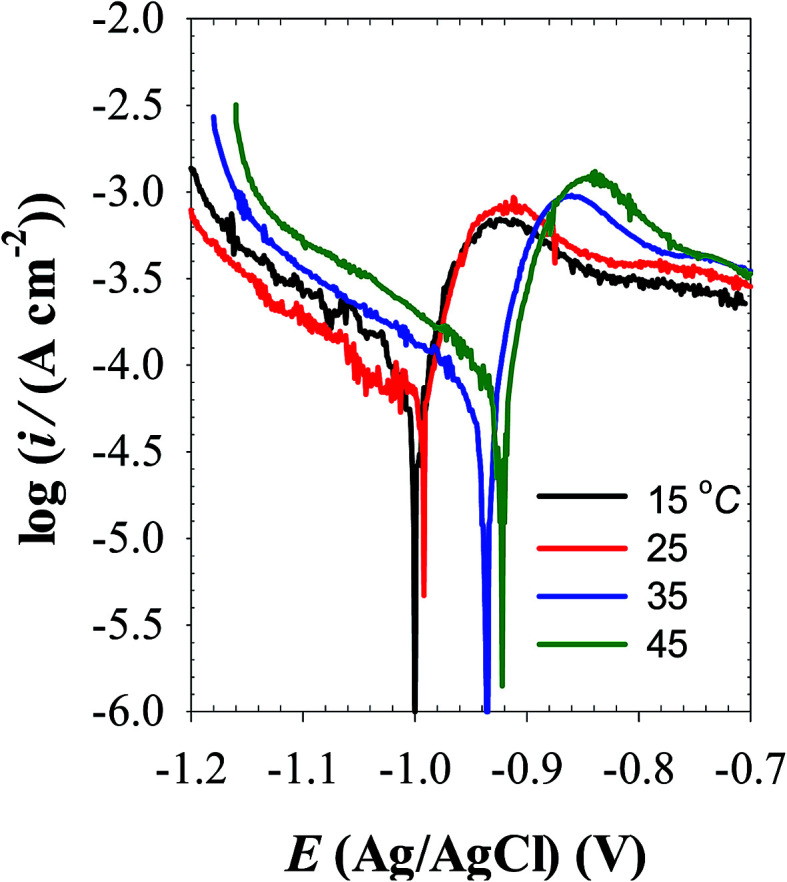
Polarization curves of Zn electrode in pH 9.8 buffer solutions at different temperatures.

Over the temperature range 5 to 25 °C in pH 9.8 buffer solution, a broadly similar cathodic and anodic polarization curves were obtained (Fig. 10) indicating similar reaction mechanism. At each temperature the polarization corrosion parameters were evaluated from those plots and compiled in Table 5. The results show that as the temperature is raised, *E*_corr_ shifts in the positive direction while *i*_corr_ increases indicating that the corrosion process is under anodic control. To further understand the mechanism of the corrosion process, the apparent activation energy (*E*_a_) of the process in pH 9.8 buffer solution was calculated using the following familiar Arrhenius eqn (11):

11 log *i*_corr_ = constant − *E*_a_/2.303*RT*

**Table 5:** Polarization corrosion parameters of zinc electrode in buffer solution of pH 9.8 at different temperatures

*T* (°C)	*E*_corr_ (V_Ag/AgCl_)	*i*_corr_ (μA cm^−2^)	*β*_a_ (mV dec^−1^)	*−β*_c_ (mV dec^−1^)
5	−1.010	15.6	32.5	117.1
15	−0.999	17.9	33.5	154.4
25	−0.996	29.4	33.6	126.4
35	−0.940	58.9	37.3	197.1
45	−0.926	90.9	49.7	218.8

**Fig. 11:**
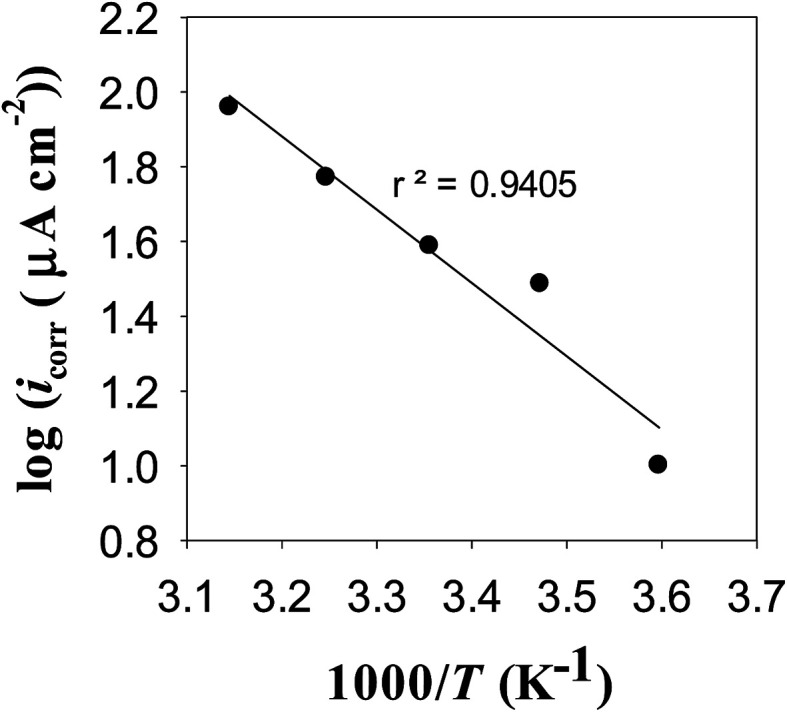
log *i*_corr_–1/*T* Arrhenius plot for pure zinc in pH 9.8 buffer solution.

The *E*_a_ value for the corrosion process is 37.91 kJ mol^−1^ as evaluated from the slope of the linear relationship between log *i*_corr_*vs.* (1/*T*) presented in Fig. 11. This value of activation energy provides an argument in favor of a solid-state diffusion model^37^ for zinc corrosion in carbonate/bicarbonate buffer solution.

### 3.2 Semiconducting properties

It is well established that passive films of metals are mainly made up of their oxides or hydroxides which are envisioned as semiconductors mainly due to the presence of native point defects (vacancies, interstitials and anti sites) in their solid structures.^38^ The electrical properties of those films are expected to be of paramount importance in understanding their protective characters against corrosion.^39^

#### 3.2.1 Mott–Schottky analysis

**Fig. 12:**
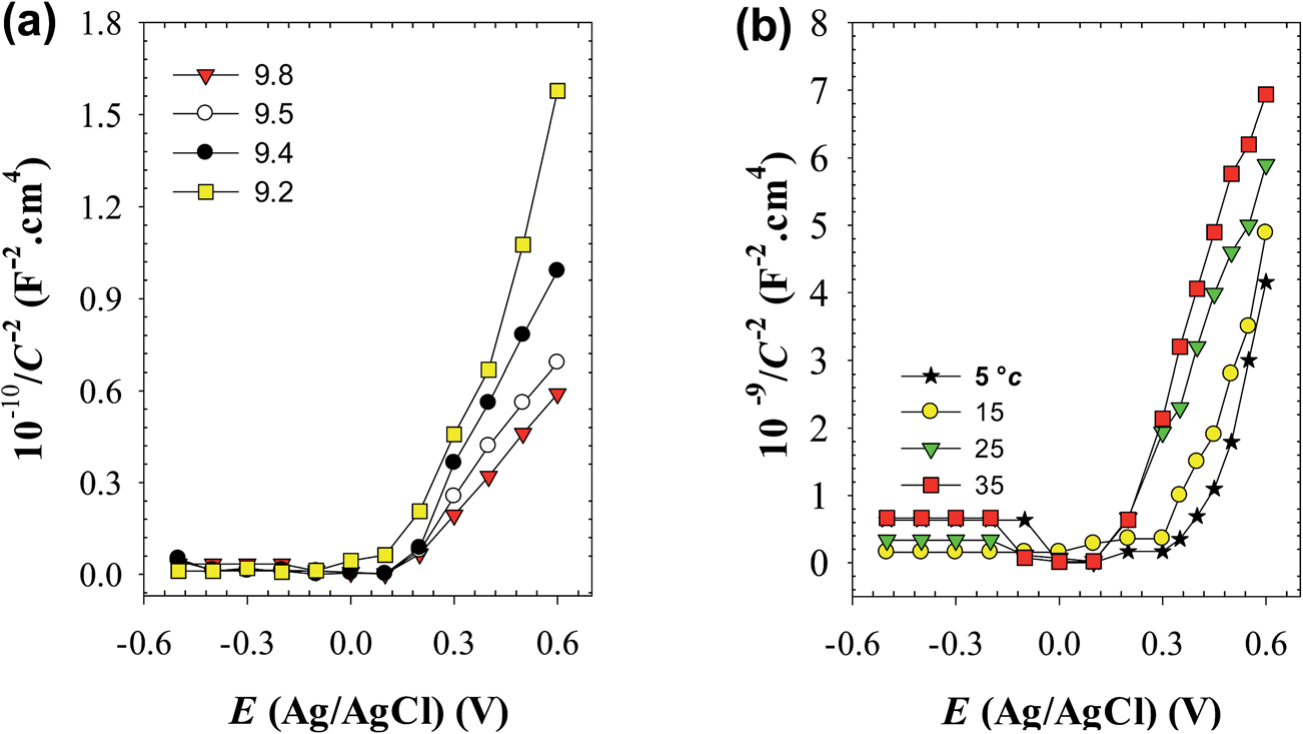
Mott–Schottky plots for the passive film on pure zinc (a) in buffer solutions of different pH values at 25 °C, and (b) in pH 9.8 buffer solution at different temperatures.

**Fig. 13:**
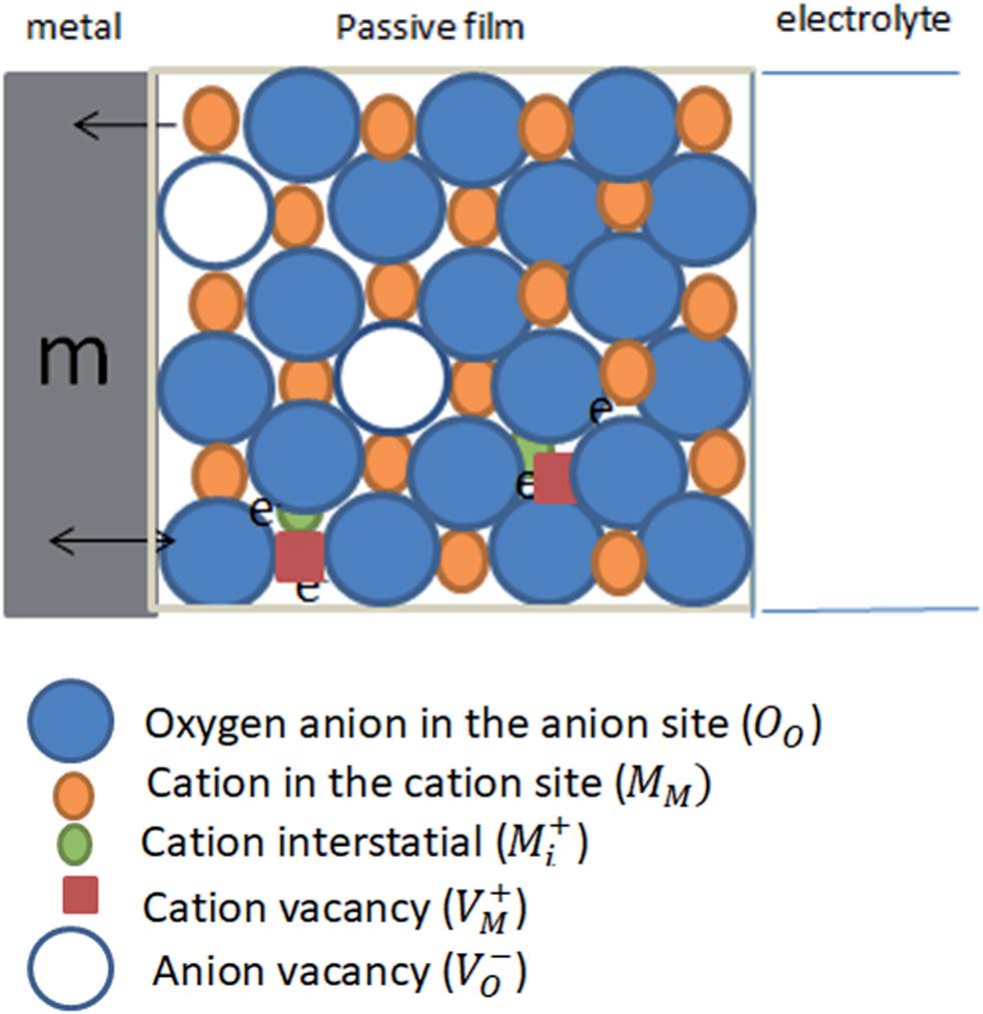
Schematic representation for the intrinsic defects present in the passive film on pure zinc metal (m).

Mott–Schottky (MS) analysis has been commonly used to examine the semiconducting properties of the passive films.^40–42^ In this series of experiments the charge distribution at the passive film/solution was acquired based on Mott–Schottky relationship by measuring electrode capacitance (*C*) *vs.* electrode potential (*E*), as a function of pH and temperature of the forming buffer solution. Fig. 12a and b shows, respectively, the MS plots for the passive films formed on pure zinc at 25 °C in buffer solutions with different pH values and in pH 9.8 buffer solution at different ambient temperatures. Generally, it can be noted that both pH and temperature of the test solution have significant influence on the electronic properties of the formed passive films.^11,19,43,44^ Over the potential range studied, the relationship between *C*^−2^ and *E* gives linear plots with positive slope under all conditions, which confirms the well-known n-type character of the passive film on zinc. The value of this slope is very dependent on the prevailing pH and temperature of the formation medium. The absence of any evidence for p-type semiconducting behavior concomitantly satisfies the criteria for both oxygen anion vacancies (V_O_^−^) and cation interstitials (M_i_^+^) to be the dominant crystallographic defects in passive layer over metal cation vacancies (V_M_^+^) and oxygen anion interstitial (O_i_^−^)^41,45,46^ as presented schematically in Fig. 13. This is probably due to the presence of free charge carriers (electrons) from the oxidative ejection of Zn^2+^ at the metal/passive film interface. In addition, it has been suggested that oxygen vacancies are responsible for the non-stoichiometry because of their relative low formation energy under oxygen poor chemical potential conditions. But oxygen vacancies are very deep donors and thus cannot be the major source of carrier electrons in n-type electrical conductivity of the passive zinc oxide. On the other hand, the zinc interstitials are shallow donors, but have high formation energy in the n-type zinc oxide.^47^ Therefore, one can suggest a preference for the native donor-type defects (V_O_^−^ and M_i_^+^) over acceptor-type defects (V_M_^+^ and O_i_^−^) in zinc oxide film.

**Fig. 14:**
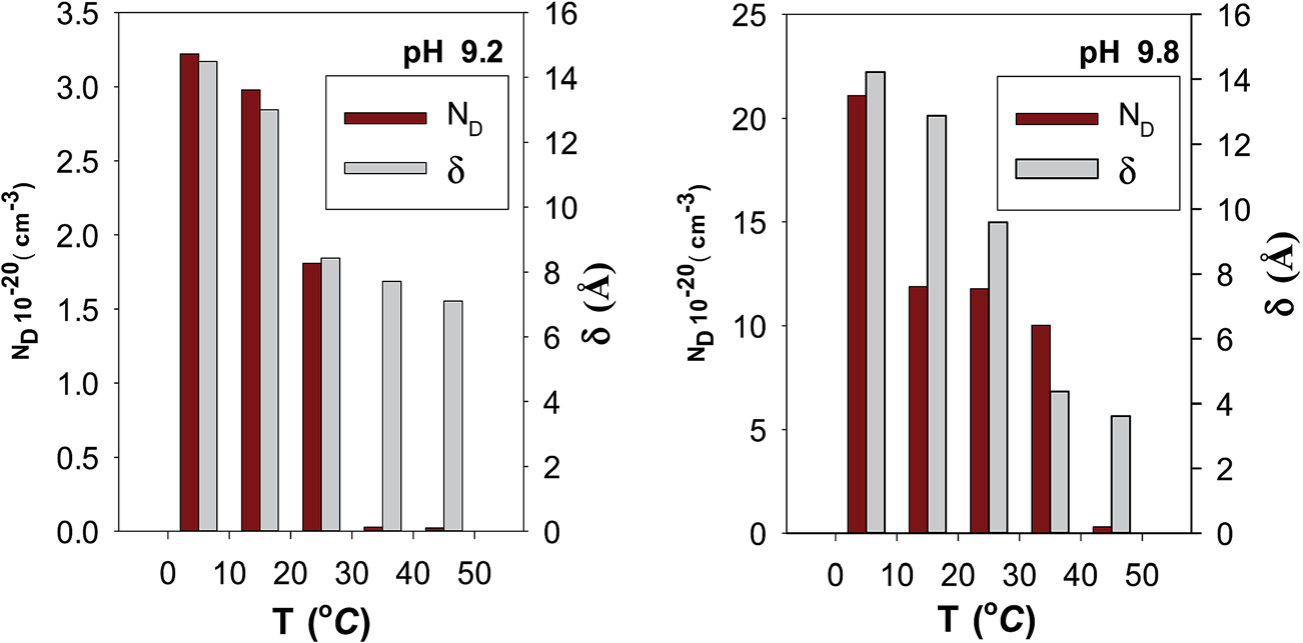
Bar diagrams of donor concentration (*N*_D_) and thickness (*δ*) for the passive film on pure zinc.

To analyze the obtained MS data, the number of defect density or donor concentration (*N*_D_) can be derived from the gradient (d*C*^−2^/d*E*) of the MS plot, and the intercept of the MS plots with the potential axis yields the flat band potential (*E*_FB_), as described in Section 2.1 based on the aforementioned eqn (1) there, and the parameters so obtained are listed in Tables 6 and 7. The results in Table 6 reveal continuous increase in the negative value of *E*_FB_ with the increase in the alkalinity of the medium, being −0.170 V at pH 9.2 and reaching −0.282 V at pH 9.8. This trend indicates lowering in the overpotential for the charge transfer reaction at the semiconductor passive film/electrolyte interface and suggests a more facile film growth on zinc surface in the most basic buffer medium^5,16,27^ in good agreement with the OCP and EIS results. It is also clear that *N*_D_ value for the charge carrier donor concentration increases with increasing the solution pH (Table 6), but it decreases with the temperature rise^39^ (Table 7). This could be correlated to a change in the film thickness (*δ*), being increased with pH and decreased with the increment of formation temperature, as proven from the values of the film thickness (*δ*) estimated from the impedance data using eqn (4) and illustrated by the bar diagram in Fig. 14.

**Table 6:** Mott–Schottky analysis for the oxide film formed spontaneously on Zn electrode as a function of the solution pH at 25 °C

pH	*V*_FB_ (V)	*N*_D_ (cm^−3^)	*δ* (Å)
9.2	−0.017	1.81 × 10^20^	8.44
9.4	−0.040	1.97 × 10^20^	8.97
9.5	−0.077	2.29 × 10^20^	9.40
9.8	−0.282	11.80 × 10^20^	9.59

**Table 7:** The donor density (*N*_D_) and the film thickness (*δ*) for the oxide film formed spontaneously on Zn electrode in buffer solutions of pH 9.8 and 9.2 at different temperatures

*T* (°C)	*N*_D_ (cm^−3^)	*δ* (Å)
**pH 9.8**		
5	2.11 × 10^21^	14.22
15	1.19 × 10^21^	12.88
25	1.18 × 10^21^	9.59
35	1.00 × 10^21^	4.36
45	3.35 × 10^19^	3.62

**pH 9.2**		
5	3.22 × 10^20^	14.49
15	2.98 × 10^20^	13.01
25	1.81 × 10^20^	8.44
35	2.68 × 10^18^	7.72
45	2.26 × 10^18^	7.11

In this connection, it has also to note that the doping donor density (*N*_D_) arises due to the highly disorder nature of the passive film and is related to the thickness of the space charge region, *i.e.* the width of the depletion layer (*δ*_SC_) *via* the following equation:^48^*δ*_SC_ = *δ*_0_(*E* − *E*_FB_)^1/2^, where *δ*_0_ = (2*εε*_0_/*qN*_D_)^1/2^. A further simplification can be made by noting that *δ*_SC_ = *δ*/*C*_SC_. It follows that calculated values of *δ*_SC_ as a function of solution pH or temperature would give the same trend as the film thickness (*δ*) *vs.* pH at constant *T* (Fig. 5a) or *δ vs. T* at constant pH value (Fig. 14). This implies that the thickness of the semiconductor passive film on zinc is a linear function of both pH and temperature of the forming medium. Furthermore, the decrease in the film resistance (*R*_f_) (Table 4) and charge carrier density (*N*_D_) (Table 7) of the passive film with raising temperature confirms the involvement of thermodynamically active mechanism in the semiconductor film grown spontaneously on zinc which leads to a subsequent reduction in its protective efficacy against corrosion.^39^

It is worth also to note that the point defect model (PDM) for the passive state^49,50^ postulates that on a pure metal, the barrier layer is basically a highly doped defect semiconductor, as demonstrated by Mott–Schottky analysis. Normally, cation vacancies are electron acceptors thereby doping the barrier layer p-type, whereas oxygen vacancies and metal interstitials are electron donors resulting in n-type doping. Thus, for zinc metal the defect structure of the passive layer can be understood in terms of the set of defect generation and annihilation reactions occurring at both the metal/passive film interface and the passive film/solution (outer layer) interface.^51,52^ The PDM further postulates that the flux of oxygen vacancy and/or cation interstitials through the passive film is essential to the film growth process. Yet, on the measured diffusivity value based on PDM, it is not possible to separate the contribution of oxygen anion vacancies and cation interstitials which are considered to be the dominant point defects in the zinc passive film. Therefore, the diffusivity is considered as that for the combination of these two intrinsic point defects acting as electron donors.

## 4. Conclusions

The influence of pH and temperature on the spontaneous passivation of Zn in alkaline carbonate/bicarbonate buffer solutions in the pH range 9.2 to 9.8 has been investigated by electrochemical measurements, Mott–Schottky analysis, FE-SEM micrographs and EDX spectra. The following conclusions can be drawn from the obtained results:

(1) Under open circuit conditions, the propensity of pure zinc to passivate was found to increase continuously with increasing the contact electrolyte pH from 9.2 to 9.8 and thus enhancing its corrosion resistance.

(2) The resistance (*R*_f_) of spontaneously formed passive film and its charge transfer resistance (*R*_ct_) are both increased with increasing solution pH, while their corresponding capacitance values *C*_f_ and *C*_dl_ are decreased. This indicates an increase in the passive film thickness (*δ*) with the increment in the forming solution pH, since *δ* value is inversely related to the film capacitance *C*_f_.

(3) During potentiodynamic polarization, transition from active to passive potential range is associated with low current density due to formation of a protective ZnO film. In pH 9.8 buffer solution, the corrosion current density over the temperature range 5 to 45 °C directly obeys Arrhenius equation and the apparent activation energy for the corrosion process was evaluated to be *E*_a_ = 37.91 kJ mol^−1^, indicating a solid state diffusion controlling mechanism.

(4) In pH 9.2 the passivation tendency similarly decreases with temperature rise up to 25 °C, but for higher temperatures (> 25 °C) the tendency of Zn to passivate was found to re-increase once more. This peculiarity was discussed and confirmed by FE-SEM and EDX examination.

(5) Mott–Schottky analysis shows that donor concentration (*N*_D_) in the surface passive film on zinc increases with increasing pH which mirror completely the trend of increasing its thickness. Additionally, the film was found to be thinner and the donor concentration be lower as the ambient temperature is raised.

## Conflicts of interest

There are no conflict of interest to declare.

## Supplementary Material
